# The Sicilian Web plAtform for Rheumatology Network: complete regional implementation and first-year real-world drug utilisation

**DOI:** 10.3389/fdgth.2026.1808227

**Published:** 2026-08-19

**Authors:** Salvatore Corrao, Giuseppe Provenzano, Mario Bentivegna, Alessandro Oteri, Pasquale Cananzi, Rita Rielli, Fabio Cartabellotta, the SWaNetwork-group

**Affiliations:** 1 Department of Internal Medicine, National Relevance and High Specialization Hospital Trust, ARNAS Civico Di Cristina Benfratelli, Palermo, Italy; 2 Department of Health Promotion Sciences, Maternal and Infant Care, Internal Medicine and Medical Specialties (PROMISE), University of Palermo, Palermo, Italy; 3 Rheumatology Unit, “Villa Sofia-Cervello” Hospital, Palermo, Italy; 4 Integrated Reference Centre of Rheumatology, ASP 7, Scicli Hospital, Ragusa, Italy; 5 Service 7 – Pharmaceutical Affairs, Department for Strategic Planning Sicily Region, Palermo, Italy; 6 Cineca Interuniversity Consortium, Bologna, Italy; 7 Department of Internal Medicine, Ospedale Buccheri La Ferla – Fatebenefratelli, Palermo, Italy

**Keywords:** implementation science, real-world drug utilisation, regional digital infrastructure, regional web-based rheumatology network, Sicilian health system

## Abstract

**Objectives:**

In the evolving landscape of chronic disease management, digital platforms have emerged as tools to support care coordination, standardised prescribing and clinical governance. Building on the precedent of the HCV Sicily Network, the Sicilian Web plAtform for Rheumatology Network (SWaNetwork) was developed to provide a regional digital infrastructure for standardised rheumatologic prescribing, conceived as the foundation of a continuously evolving regional real-world data ecosystem. This report describes the complete regional implementation of the SWaNetwork across all authorised Sicilian rheumatology prescribing centres and presents the first-year real-world prescribing data generated by the platform.

**Methods:**

SWaNetwork is a secure, web-based platform launched in October 2022, designed to manage the complete process of prescribing and dispensing rheumatologic therapies. Developed in collaboration with the CINECA Interuniversity Consortium, the system integrates data from 14 regional prescribing centres, providing real-time dashboards, automated alerts, and structured reports to support clinical governance. Descriptive analyses were performed using Stata 18. This is a descriptive implementation and drug-utilisation report; no comparator group or clinical-effectiveness outcomes were analysed.

**Results:**

In its first year, all 14 authorised regional prescribing centres were fully onboarded and 71 healthcare professionals registered 8,243 patients, with a platform-defined follow-up completion rate of 98%. A total of 1,426 initial prescriptions and 40,025 follow-up prescriptions were recorded (41,451 prescriptions overall), predominantly involving biologics such as Adalimumab (33.4%) and Etanercept (24.3%). These findings describe the scale and drug-utilisation pattern generated during the first year of regional implementation.

**Conclusions:**

The SWaNetwork achieved complete regional implementation and generated a comprehensive first-year real-world prescribing dataset across Sicily. Rather than demonstrating clinical effectiveness, this foundational report describes a regional digital infrastructure for standardised prescribing governance, laying the foundation for a continuously evolving regional real-world data ecosystem, and provides the basis for future studies on treatment persistence, drug survival, switching, effectiveness, safety, healthcare utilisation and pharmacoeconomic outcomes.

## Graphical Abstract

**Figure:**
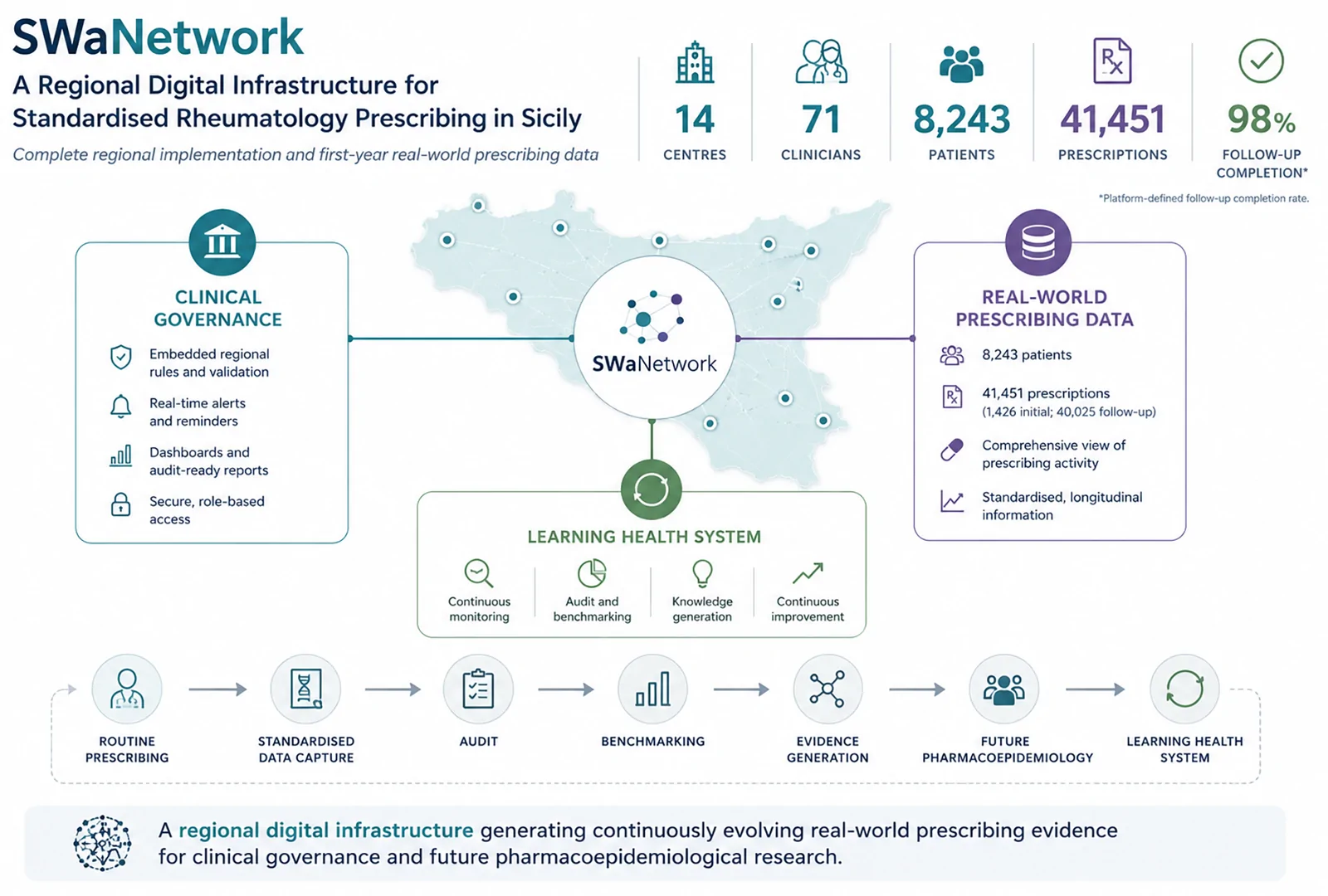

## Introduction

Digital health is progressively evolving from isolated, single-purpose registries towards continuously learning regional digital infrastructures that generate structured real-world information as an intrinsic by-product of routine care. Digital health infrastructures are increasingly used to support regional clinical governance, particularly in therapeutic areas characterised by high treatment complexity and cost. The management of inflammatory rheumatic diseases relies heavily on biologic and targeted synthetic disease-modifying antirheumatic drugs (b/tsDMARDs), whose expanding use, high per-patient cost and requirement for structured eligibility and monitoring create a strong rationale for standardised, regionally coordinated prescribing workflows. Web-based platforms that centralise patient registration, prescribing and dispensing information can embed such standardisation directly into clinical practice, enabling systematic data capture, audit-ready reporting and a shared regional overview of prescribing activity. Within this evolving landscape, a platform of this kind is best understood not merely as prescribing software, but as a digital health infrastructure with a cumulative and expanding evidentiary role.

In Sicily, the growing number of authorised prescribing centres and the complexity of regional reimbursement rules for b/tsDMARDs created the need for a single digital infrastructure capable of harmonising prescribing practice across the region, supporting regional health authorities in monitoring regional prescribing activity and expenditure, and providing a shared platform for clinicians, pharmacies and regional decision-makers (1).

In recent years, to tackle the challenges posed by chronic hepatitis C virus and its new therapies, the HCV Sicily Network was developed as a web-based model of best medical practice to optimise managing and treating chronic hepatitis C and cirrhosis. This innovative network encompasses 41 centres and connects 84 gastroenterologists and Infectious disease specialists through a centralised web platform, facilitating the recording of diagnoses and treatment priorities.

From March 2015 to September 2016, the network documented 9,965 patients (57% male, mean age 61 years, with 34% aged over 70 years), of whom 3,319 completed treatment and 1,754 underwent a 12-week follow-up. The network reported a Sustained Virological Response (SVR) in 1,541 patients (87.8%), while 136 patients (7.7%) and 77 patients (4.5%) experienced virological relapses during the follow-up period. This real-world experience provides evidence of robust results from randomised clinical trials in clinical practice and serves as an example of effective resource utilisation (2).

The experience of the HCV Sicily Network demonstrated its potential as a model of regional digital infrastructure for the Regional Department of Health, supporting standardised regional management of complex, high-cost therapies (2). This model inspired the “Sicilian Web plAtform for Rheumatology Network (the SWaNetwork)”, designed to provide an analogous centralised digital infrastructure for the regional management of rheumatologic prescribing. In this sense, the SWaNetwork was conceived not as a prescribing software tool but as a regional digital health infrastructure intended to support standardised care over time.

This manuscript is the foundational report of the SWaNetwork, describing the complete regional implementation of the platform and presenting the first-year real-world prescribing data it generated across Sicily; this scope purposefully stops short of evaluating treatment effectiveness or clinical outcomes.

## Methods

### Study design

This is a descriptive, retrospective analysis of implementation and real-world drug-utilisation data generated by the SWaNetwork platform during its first year of operation (October 2022 – September 2023). The study did not include a comparator group and was not designed to evaluate clinical effectiveness, guideline adherence, administrative burden or cost-effectiveness.

### Platform architecture and workflow

The analysed data originates from the SWaNetwork platform, which manages medication prescription and dispensing processes within Sicily's rheumatology sector. Developed in collaboration with the Interuniversity Consortium CINECA, this platform has been operational since October 2022 for authorised regional centres. It enables the standardised and integrated management of the entire information flow, from the centralised registration of patients with rheumatic diseases to prescription of medications by authorised physicians and, ultimately, their dispensing by pharmacies, thereby establishing a network among the various stakeholders involved.

By establishing a centralised regional database, the SWaNetwork enables continuous monitoring of patients' clinical pathways related to rheumatic diseases and facilitates patient transfers between centres without duplicating information.

### Regional implementation

The platform was rolled out progressively across all 14 authorised rheumatology prescribing centres in Sicily, achieving complete regional coverage by the end of the first year of operation. Onboarding involved registration of authorised prescribing physicians and pharmacy staff, configuration of centre-specific access profiles, and integration of each centre into the shared regional database.

### Embedded prescribing governance

The platform incorporates regional prescribing rules into the digital workflow through mandatory fields, predefined pathways, automated validation checks, alerts and hard-stop controls governing eligibility, dosing and monitoring requirements for b/tsDMARDs. Prescriptions are therefore generated within a rule-based regional governance environment. The present study does not independently measure guideline adherence as an outcome; rather, it describes the drug-utilisation output produced within this governance framework.

### Dashboard, alerts and reports

The SWaNetwork provides interactive, role-based dashboards accessible to clinicians, pharmacy staff and regional health authorities. At centre level, the dashboard displays the number of registered patients, active prescriptions, initial and follow-up prescriptions, drug-utilisation distribution, dispensing status, and outstanding or incomplete mandatory fields. At regional level, aggregate reports summarise prescribing volumes, drug distribution and platform-defined follow-up completion across all participating centres. Automated functions include prescription-renewal reminders, mandatory-field alerts that prevent prescription completion when eligibility or monitoring information is missing, validation checks against standardised drug and diagnosis dictionaries, and audit-ready reports intended to support periodic regional review of prescribing activity.

Representative screenshots were not included in this manuscript because of institutional data-protection and platform-confidentiality constraints; the main dashboard functions and automated controls are nonetheless described here in full.

### Data quality procedures

Delivered as web-based Software as a Service (SaaS), the platform ensures secure and protected access to information tailored for different user profiles and access levels. Automated control procedures during the data input stage, which include real-time alerts and warnings and the use of standardised dictionaries, uphold data quality.

### Implementation indicators

Implementation was described using the number of participating prescribing centres, the number of registered healthcare professionals, and the number of patients registered on the platform during the first year of operation.

### Drug-utilisation indicators

Drug utilisation was described using the number of initial and follow-up prescriptions and the distribution of prescribed biologic and targeted synthetic DMARDs (b/tsDMARDs), expressed as absolute frequencies and percentages.

### Follow-up definition

Follow-up completion was defined according to the platform-specific follow-up field recorded in the SWaNetwork database, which is populated when a scheduled follow-up prescription or monitoring record is completed within the platform. This indicator should therefore be interpreted as a measure of completeness of platform-recorded follow-up information rather than as a clinical outcome such as treatment persistence or continuity of care.

### Statistical analysis

Stata Statistical Software (Release 18, College Station, TX: StataCorp LLC; 2023) was used to perform all the descriptive analyses, computing absolute and relative frequencies.

## Results

**Figure 1:**
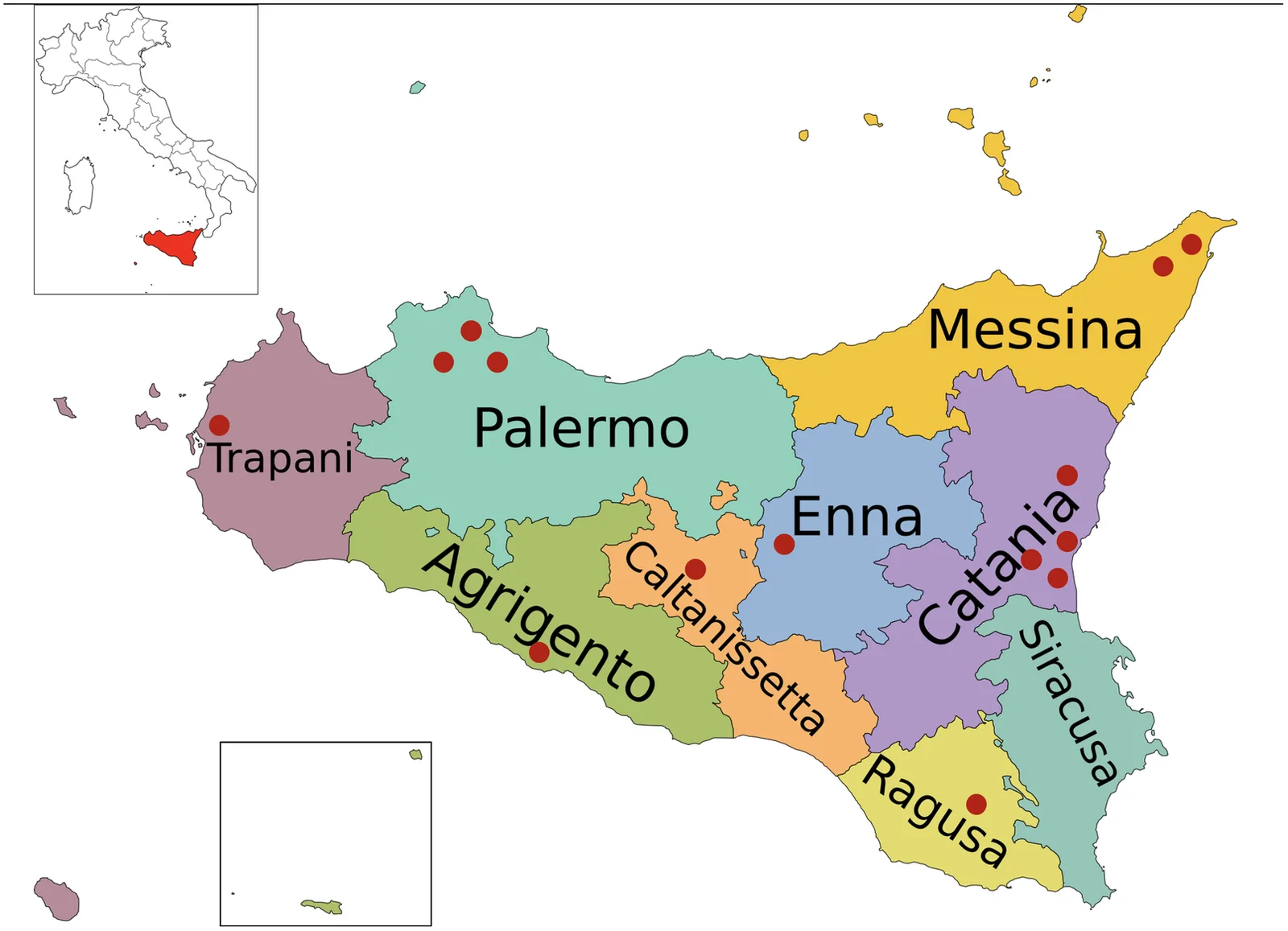
Geographical distribution of rheumatology prescribing centres within the Sicilian Web Platform for Rheumatology Network (SWaNetwork). The red dots represent the accredited prescribing centres in Sicily's provinces, ensuring widespread access to specialised rheumatologic care. Map from “Map of region of Sicily, Italy, with provinces” by Vonviken.

During the first year of operation, the SWaNetwork achieved complete regional implementation, with all 14 authorised prescribing centres onboarded and 71 healthcare professionals registered on the platform, managing care for 8,243 patients across the 14 centres. Figure 1 shows the geographical distribution of the prescribing centres across Sicily's provinces.

The present analysis reports implementation and drug-utilisation data at regional level. Centre-level comparative analyses were not included because the objective of this foundational report was to document regional deployment and overall drug-utilisation output rather than to compare individual centres.

The platform replaced fragmented local prescription documentation with a unified regional digital workflow shared by clinicians, pharmacies and regional healthcare authorities.

**Figure 2:**
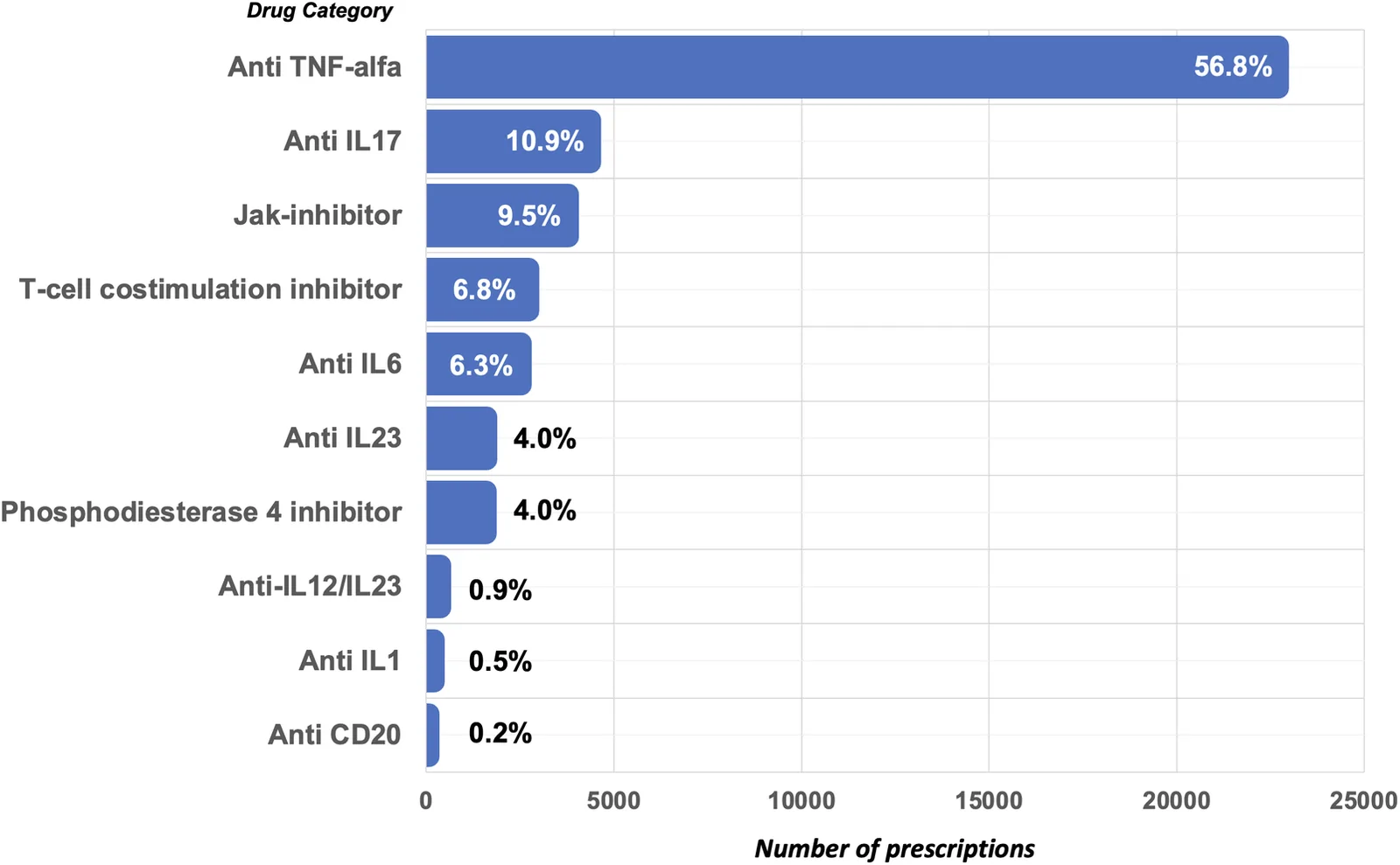
Distribution of prescriptions recorded in the SWaNetwork database, the rheumatology registry of the Sicilian network. The horizontal bar chart illustrates the absolute values and percentages of prescriptions for biologic and targeted synthetic disease-modifying antirheumatic drugs (b/tsDMARDs). *X*-axis indicates the number of prescriptions, with rates for each category displayed within the bars, while *y*-axis lists the drug categories.

Among registered patients, the platform-defined follow-up completion rate was 98%. Initial prescriptions (*n* = 1,426) prominently included biologic agents, such as Adalimumab (33.4%) and Etanercept (24.3%). Follow-up prescriptions (*n* = 40,025) were generated within the platform-embedded regional prescribing framework, which requires mandatory eligibility and monitoring information before prescription completion (41,451 prescriptions overall). The platform provides longitudinal prescription information that may support therapeutic reassessment during routine clinical practice. Figure 2 shows the distribution of biologics recorded on the platform.

## Discussion

### Main finding

The principal finding of this study is that the SWaNetwork achieved complete regional implementation across all 14 authorised rheumatology prescribing centres in Sicily within its first year of operation, and generated a substantial first-year real-world prescribing dataset comprising 8,243 registered patients, 1,426 initial prescriptions and 40,025 follow-up prescriptions. These findings describe the scale and reach of the regional digital infrastructure rather than its clinical impact. Beyond its immediate descriptive value, the significance of this finding lies in the fact that complete regional implementation establishes a continuously operating regional digital ecosystem, whose principal scientific contribution is not the prescribing figures reported here but the durable capacity to generate structured real-world information for clinical governance and future research.

### Meaning of complete regional implementation

Achieving full coverage of all authorised prescribing centres within one year indicates that a shared regional digital workflow for rheumatologic prescribing is operationally feasible across a heterogeneous network of academic, general and community hospitals. This regional scale distinguishes the SWaNetwork from single-centre or pilot digital-health initiatives and provides the coverage required for a representative regional prescribing dataset.

An additional, often underappreciated implication of complete regional coverage concerns representativeness. Because the SWaNetwork captures prescribing activity from every authorised centre rather than from a self-selected subset, the resulting database reflects routine prescribing across an entire regional healthcare system rather than the practice of early-adopting or academically oriented centres alone. This population-scale coverage is likely to reduce provider-level selection bias relative to registries that rely on voluntary participation, and should improve the representativeness of future observational analyses derived from this infrastructure; we present this consideration cautiously, since coverage at the prescribing-centre level does not, by itself, guarantee representativeness at the patient level.

### SWaNetwork as a digital prescribing infrastructure

The observed distribution of biologic therapies, dominated by Adalimumab and Etanercept, is broadly consistent with prescription patterns reported in previous studies (3). Because prescriptions are generated within a rule-based environment that embeds regional eligibility and monitoring requirements, this distribution should be interpreted as the real-world prescribing output of a governed regional workflow rather than as independent evidence of guideline adherence, which was not directly measured in this study.

The platform's interactive dashboards and predefined reports provide regional stakeholders with continuous visibility of prescribing trends, replacing the fragmented, centre-level data collection that preceded the SWaNetwork. Whether this visibility translates into detection and correction of deviations from standard practice, as suggested by the broader audit-and-feedback literature (4, 5), remains a specific target for future evaluation.

This distinction matters conceptually. Unlike traditional registries, which typically depend on dedicated, resource-intensive data-entry efforts superimposed on clinical work, a digital prescribing infrastructure such as the SWaNetwork generates structured real-world information as an intrinsic by-product of routine prescribing. Because data capture is embedded in care delivery rather than added to it, the resulting evidence base is expected to accumulate continuously and to grow in scientific value as successive years of observation are added, rather than representing a fixed, one-time dataset.

### Towards a learning regional health system

Consequently, the scientific value of the SWaNetwork lies not only in the volume of prescribing data collected, but in the continuous interaction between routine prescribing, regional governance and knowledge generation, whereby each prescribing cycle incrementally enriches the regional evidence base. The architecture of the SWaNetwork can be situated within the broader concept of the learning health system (6), in which routinely collected clinical data are continuously translated into knowledge that, in turn, informs and improves practice. In this framework, the continuous acquisition of regional prescribing information supports continuous audit of prescribing patterns against regional pathways; continuous audit enables continuous benchmarking across prescribing centres; continuous benchmarking creates the conditions for continuous learning about regional practice variation; and continuous learning, in principle, supports continuous optimisation of regional prescribing governance. We present this conceptual trajectory cautiously: the present report demonstrates only the first step of this cycle, namely the continuous acquisition of regional prescribing information, and does not claim to have demonstrated audit, benchmarking, learning or optimisation effects.

### Embedded regional prescribing governance

Rather than measuring guideline adherence as an outcome, the SWaNetwork embeds regional prescribing rules directly into the prescribing workflow through mandatory fields, predefined pathways and hard-stop validation checks. This design shifts governance upstream, to the point of prescribing, rather than relying solely on retrospective audit. The real-world prescribing data presented here should therefore be understood as the output of this governed workflow rather than as an independently measured adherence outcome.

### First-year real-world prescribing output

The first-year dataset — 8,243 patients, 1,426 initial and 40,025 follow-up prescriptions — constitutes, to our knowledge, the first regional-scale, platform-generated prescribing dataset for rheumatologic b/tsDMARD prescribing in Sicily. Its principal value lies not in demonstrating a clinical effect but in establishing a reproducible, structured data source that can support future longitudinal analyses, and in laying the first layer of what is expected to become an increasingly comprehensive regional real-world prescribing ecosystem as subsequent years of data accumulate.

### Audit and clinical governance potential

The infrastructure created by the SWaNetwork provides the technical basis for periodic clinical audit and continuous quality-improvement cycles, in line with the broader literature on audit-and-feedback interventions in professional practice (7). Structured audit cycles built on this platform could, in principle, evaluate adherence to diagnostic and therapeutic pathways and support evidence-based adjustment of prescribing strategies (8); representing a natural next application for this infrastructure rather than an analysis undertaken as part of the present report.

Beyond its potential relevance to individual clinicians, this infrastructure is, in principle, equally relevant to regional healthcare authorities, for whom it may support resource planning, facilitate evaluation of prescribing policy, enable ongoing surveillance of regional prescribing patterns, and create the conditions for monitoring the uptake of therapeutic innovation. We describe these as potential applications of the infrastructure rather than as benefits demonstrated by the present study.

The dashboards also support the generation of regional benchmarking reports that could, in future work, enable performance comparisons across centres (9). As noted above, centre-level comparison lies outside the scope of this foundational, regional-level report.

### Foundation for future pharmacoepidemiological studies

Building on this governance potential, the principal value of the SWaNetwork lies in the creation of a regional longitudinal real-world database. Large longitudinal digital infrastructures of this kind tend to evolve, over successive years of operation, from single implementation reports into regional research ecosystems, whose informativeness for real-world evidence generation increases cumulatively as further prescribing cycles, outcomes and follow-up data are captured. The present report should be considered the foundational description of the enabling digital infrastructure rather than an evaluation of clinical outcomes. Future dedicated analyses based on the SWaNetwork database will be able to address treatment persistence, drug survival, switching patterns, comparative effectiveness, safety, predictors of response, healthcare utilisation and pharmacoeconomic impact.

### Limitations

Several limitations should be acknowledged. First, this was a descriptive implementation and real-world prescribing study without a comparator group; therefore, no causal inference can be made regarding clinical outcomes, administrative burden, treatment effectiveness or cost-effectiveness. Second, adherence to regional prescribing requirements was not independently measured as an outcome; rather, these requirements were embedded into the platform workflow. Third, the reported follow-up completion rate depends on the platform-defined follow-up field and should be interpreted accordingly, rather than as a measure of clinical continuity of care. Fourth, more granular centre-level comparisons and longitudinal analyses of persistence, effectiveness, switching and safety require dedicated methodological designs and fall outside the scope of the present foundational report. Finally, the data presented cover only the first year of platform operation and may not capture longer-term patterns of use.

Additionally, while the platform has achieved complete implementation in Sicily's regional context, its applicability in other regions or countries with different healthcare structures would need further investigation. Customisation and adaptability will be crucial factors in determining whether similar platforms can be effectively implemented elsewhere.

Integrating patient-reported outcomes and quality-of-life measures into the platform could, in future dedicated studies, offer a more thorough evaluation of treatment effectiveness from the patients' perspective. Including this data could further refine treatment plans and improve shared decision-making between patients and providers .

## Conclusion

Rather than representing the endpoint of a digital-health project, the SWaNetwork should be regarded as the beginning of a continuously evolving regional digital infrastructure, having already achieved complete regional implementation across authorised rheumatology prescribing centres in Sicily and generated a comprehensive first-year real-world prescribing dataset. By embedding regional prescribing rules within a shared digital workflow, the platform establishes a regional infrastructure for standardised prescription governance and audit-ready monitoring. Subsequent analyses evaluating treatment persistence, drug survival, switching patterns, comparative effectiveness, safety, healthcare utilisation and pharmacoeconomic outcomes will progressively extend this evidence base, whose scientific value — in keeping with the logic of a learning regional health infrastructure — is expected to grow with every successive year of accumulated real-world data.

## Data Availability

The raw data supporting the conclusions of this article will be made available by the authors, without undue reservation.

## Group member of the SWaNetwork-group investigators

Agata Domenica La Rosa - Farmacia (A.O. Cannizzaro), Anna Nicolosi - Farmacia (A.O. Cannizzaro), Antonella Di Maggio - Farmacia (A.O. Cannizzaro), Cesarina Montera - Farmacia (A.O. Cannizzaro), Gabriella Petralia - Farmacia (A.O. Cannizzaro), Gianluca Sambataro - Clinico (A.O. Cannizzaro), Michele Colaci - Clinico (A.O. Cannizzaro), Teresa Travaglianti - Farmacia (A.O. Cannizzaro), Alberto Lo Gullo - Clinico (A.O. Papardo), Aldo Molica Colella - Clinico (A.O. Papardo), Flavia Rossano - Farmacia (A.O. Papardo), Maria Rosa Luppino - Farmacia (A.O. Papardo), Rossella Caminiti - Farmacia (A.O. Papardo), Aldo Palazzo - Farmacia (A.O. Villa Sofia - Cervello), Antonino Santoro - Farmacia (A.O. Villa Sofia - Cervello), Chiara Lucchese - Farmacia (A.O. Villa Sofia - Cervello), Emma Aragona - Clinico (A.O. Villa Sofia - Cervello), Gioacchino Di Liberti - Farmacia (A.O. Villa Sofia - Cervello), Giuseppe Provenzano - Clinico (A.O. Villa Sofia - Cervello), Maria Concetta Miceli - Clinico (A.O. Villa Sofia - Cervello), Piera Polidori - Farmacia (A.O. Villa Sofia - Cervello), Roberto Bursi - Clinico (A.O. Villa Sofia - Cervello), Agata Vitale - Clinico (A.O.U. Gaetano Martino), Cristina Guarnera - Farmacia (A.O.U. Gaetano Martino), Donatella Sangari - Clinico (A.O.U. Gaetano Martino), Eleonora Mocciaro - Farmacia (A.O.U. Gaetano Martino), Fabiola Atzeni - Clinico (A.O.U. Gaetano Martino), Gabriela Wasniewska - Clinico (A.O.U. Gaetano Martino), Gianfranco Miceli - Clinico (A.O.U. Gaetano Martino), Giovanni Conti - Clinico (A.O.U. Gaetano Martino), Giuseppina Rossello - Farmacia (A.O.U. Gaetano Martino), Lida Lombardo - Farmacia (A.O.U. Gaetano Martino), Nicola Pellegrino - Farmacia (A.O.U. Gaetano Martino), Roberto Chiemenz - Clinico (A.O.U. Gaetano Martino), Tiziana Genovese - Clinico (A.O.U. Gaetano Martino), Vincenzo Arcoraci - Clinico (A.O.U. Gaetano Martino), Claudia Schinocca - Clinico (A.O.U. Policlinico Paolo Giaccone), Eva De Luca - Farmacia (A.O.U. Policlinico Paolo Giaccone), Giorgio Buscetta - Clinico (A.O.U. Policlinico Paolo Giaccone), Giulia Grasso - Clinico (A.O.U. Policlinico Paolo Giaccone), Giuliana Guggino - Clinico (A.O.U. Policlinico Paolo Giaccone), Anna Schillaci - Farmacia (A.O.U. Policlinico San Marco), Carla Moscheo - Clinico (A.O.U. Policlinico San Marco), Elisa Maria Platania - Farmacia (A.O.U. Policlinico San Marco), Elisa Visalli - Clinico (A.O.U. Policlinico San Marco), Giorgio Amato - Clinico (A.O.U. Policlinico San Marco), Giuseppe Benfatto - Farmacia (A.O.U. Policlinico San Marco), Laura Terranova - Farmacia (A.O.U. Policlinico San Marco), Martino Di Pietro - Farmacia (A.O.U. Policlinico San Marco), Patrizia Barone - Clinico (A.O.U. Policlinico San Marco), Patrizia Campione - Farmacia (A.O.U. Policlinico San Marco), Roberta Foti - Clinico (A.O.U. Policlinico San Marco), Rosario Foti - Clinico (A.O.U. Policlinico San Marco), Rossana Angela Scavone - Farmacia (A.O.U. Policlinico San Marco), Serafina Cantarella - Farmacia (A.O.U. Policlinico San Marco), Ylenia Dal Bosco - Clinico (A.O.U. Policlinico San Marco), Giorgia Virzì - Clinico (ARNAS Civico Benfratelli), Giovanni Pistone - Clinico (ARNAS Civico Benfratelli), Ignazio Cangemi - Clinico (ARNAS Civico Benfratelli), Patrizia Marrone - Farmacia (ARNAS Civico Benfratelli), Salvatore Corrao - Clinico (ARNAS Civico Benfratelli), Umberto Lupo - Clinico (ARNAS Civico Benfratelli), Alessandra Azzurra Russo - Clinico (ARNAS Garibaldi), Barbara Busà - Farmacia (ARNAS Garibaldi), Cecilia Testa - Farmacia (ARNAS Garibaldi), Daniela Sindoni - Farmacia (ARNAS Garibaldi), Gesualdo Interlandi - Farmacia (ARNAS Garibaldi), Maria Ilenia De Andres - Clinico (ARNAS Garibaldi), Maria Rosa Di Benedetto - Farmacia (ARNAS Garibaldi), Roberto Mario Michel Patanè - Clinico (ARNAS Garibaldi), Alessia Benenati - Clinico (P. O. Umberto I), Maria Alba Spadafora - Clinico (P. O. Umberto I), Salvatrice Nicita - Farmacia (P. O. Umberto I), Giovanni Mario D'Avola - Clinico (P.O. Acireale), Maria Grazia Aloisi - Farmacia (P.O. Acireale), Maria Pia Salanitro - Farmacia (P.O. Acireale), Emanuele Cassara - Clinico (P.O. Busacca), Francesca Marino - Clinico (P.O. Busacca), Mario Bentivegna - Clinico (P.O. Busacca), Antonella Amato - Farmacia (P.O. S. Antonio Abate), Carmelo Debilio - Clinico (P.O. S. Antonio Abate), Cristina Consuelo Trapani - Farmacia (P.O. S. Antonio Abate), Federica Bazza - Farmacia (P.O. S. Antonio Abate), Giulia Cancellieri - Farmacia (P.O. S. Antonio Abate), Giuseppe Antonino Attaguile - Farmacia (P.O. S. Antonio Abate), Giuseppe Palermo - Farmacia (P.O. S. Antonio Abate), Irene Bongiorno - Farmacia (P.O. S. Antonio Abate), Katia Ornella Costa - Farmacia (P.O. S. Antonio Abate), Marco Cicoira - Farmacia (P.O. S. Antonio Abate), Maria Perla Clemente - Farmacia (P.O. S. Antonio Abate), Silvana Venezia - Farmacia (P.O. S. Antonio Abate), Alfonso Principato - Clinico (P.O. San Giovanni di Dio), Giuseppa Mira - Clinico (P.O. San Giovanni di Dio), Pamela Sferrazza - Clinico (P.O. San Giovanni di Dio), Fortunato Ferracane - Clinico (P.O. Vittorio Emanuele), Francesca Maria Cafà - Farmacia (P.O. Vittorio Emanuele), Gaetano Cannizzo - Farmacia (P.O. Vittorio Emanuele), Giancarlo Sillitti - Farmacia (P.O. Vittorio Emanuele), Maria Gellina - Farmacia (P.O. Vittorio Emanuele), Rosario Trainito - Farmacia (P.O. Vittorio Emanuele).
